# Extended High Frequency Audiometry in Polycystic Ovary Syndrome

**DOI:** 10.1155/2013/482689

**Published:** 2013-09-30

**Authors:** Cuneyt Kucur, Suna Kabil Kucur, Ilay Gozukara, Ali Seven, Kadriye Beril Yuksel, Nadi Keskin, Fatih Oghan

**Affiliations:** ^1^Medical School, Evliya Celebi Training and Research Hospital, Department of Otolaryngology, Dumlupinar University, Okmeydani Street, 43340 Kutahya, Turkey; ^2^Department of Obstetrics and Gynecology, Medical School, Dumlupinar University, 43340 Kutahya, Turkey

## Abstract

*Objective*. Polycystic ovarian syndrome (PCOS) is the most common endocrine disorder affecting 5–10% of women in reproductive age. Insulin resistance, dyslipidemia, glucose intolerance, hypertension, and obesity are metabolic disorders accompanying the syndrome. PCOS is a chronic proinflammatory state and the disease is associated with endothelial dysfunction. In diseases with endothelial damage, hearing in high frequencies are mostly effected in early stages. We evaluated extended high frequency hearing loss in PCOS patients. *Material Methods*. Forty women diagnosed as PCOS and 25 healthy controls were included in this study. Age and BMI of PCOS and control groups were comparable. Each subject was tested with low (250–2000 Hz), high (4000–8000 Hz), and extended high frequency audiometry (8000–20000). Hormonal and biochemical values including LH, LH/FSH, testosterone, fasting glucose, fasting insulin, HOMA-I, and CRP were calculated. *Results*. PCOS patients showed high levels of LH, LH/FSH, testosterone, fasting insulin, glucose, HOMA-I, and CRP levels. The hearing thresholds of the groups were similar at frequencies of 250, 500, 1000, 2000, and 4000 Hz; statistically significant difference was observed in 8000–14000 Hz in PCOS group compared to control group. *Conclusion*. PCOS patients have hearing impairment especially in extended high frequencies. Further studies are needed to help elucidate the mechanism behind hearing impairment in association with PCOS.

## 1. Introduction

Polycystic ovarian syndrome (PCOS) is the most common endocrine disorder affecting 5–10% of women in reproductive age [1]. The etiology is unknown. Disease is characterized by oligo-amenorrhea, hyperandrogenism, and polycystic ovaries [2]. PCOS is a chronic condition beginning most commonly in adolescence. Insulin resistance, dyslipidemia, glucose intolerance, hypertension, obesity, and nonalcoholic fatty liver are metabolic disorders accompanying syndrome [3–5]. Therefore, a higher cardiovascular risk is reported [6]. Endothelial cell dysfunction is one of the earliest stages of atherogenesis. Therefore, markers reflecting endothelial injury have been searched [7]. Tumor necrosis factor, highly sensitive C-Reactive Protein, homocysteine, and Plasminogen activator inhibitor-1 are some of the cardiovascular risk markers that are increased in PCOS [8]. PCOS is a chronic proinflammatory state. An imbalance between prooxidants and antioxidants in PCOS produces an oxidative state [9]. There is also an association between inflammation at the molecular level and insulin resistance in the disorder [8, 10]. Elevations of a number of circulating proatherogenic inflammatory mediators have been independently reported in PCOS [11, 12]. Meta-analysis of the 31 articles reported that circulating CRP was 96% higher in women with PCOS compared to healthy controls [13]. The relationship between CRP and atherothrombotic cardiovascular disease, renal function abnormalities, has been reported in a number of studies [14]. Factors predisposing for endothelial injury include hyperinsulinemia, insulin resistance, dyslipidemia, and chronic low-grade inflammation which often accompanies PCOS [15].

Recently, a study conducted by Oghan et al. showed that patients with PCOS have sensorineural hearing loss in high frequencies [16]. They postulated that hyperandrogenism was the possible etiological factor. However, to date, the relationship between PCOS and hearing has not been fully understood. The aim of the present study was to determine the status of extended high frequency audiometry in cases of PCOS and to evaluate if there are other possible contributing factors that cause hearing loss.

## 2. Material Method

The study was carried out at Dumlupinar University Kutahya Evliya Celebi Training and Research Hospital gynecology outpatient clinic. Forty patients with the PCOS and twenty-five healthy subjects were enrolled in this prospective study. The control group consisted of patients who admitted to hospital for routine gynecological examination.

We included healthy women as controls with normal menstrual cycles, with no evidence of hyperandrogenism, and with normal ovarian morphology on pelvic ultrasonography. Ferriman Gallwey scores of all control patients were under 8. PCOS was defined as the presence of two of the following three features after the exclusion of other etiologies [17], (i) oligo- or anovulation (fewer than six menstrual periods in the preceding year), (ii) hyperandrogenism and/or biochemical signs of hyperandrogenism, and/or (iii) polycystic ovaries.

The study was conducted according to the guidelines for clinical studies described in the Declaration of Helsinki (as revised by the World Medical Association, http://www.wma.net/). Regional Ethical Committee approved the study. All patients gave oral and written informed consent prior to the examination.

Exclusion criteria were tinnitus, middle ear disease, diabetes mellitus, family history of hearing loss, history of acoustic trauma, conductive hearing loss, exposure to ototoxic substances, occupational noise exposure, autoimmune diseases, history of smoking, ongoing infection-inflammation, and being on any medication.

Body mass index (BMI) was calculated as weight (kg)/height (m)^2^. Systolic (SBP) and diastolic blood pressure (DBP) were measured twice in the right arm in a relaxed sitting position. Two measurements were taken 15 minutes apart. The average of two was used.

Blood samples were collected during early follicular phase of menstrual cycle after at least 12-hour fasting. Levels of glucose, insulin, and hormone profile (follicle-stimulating hormone (FSH), luteinizing hormone (LH), estradiol (E2), total and free testosterone (Total-T and Free-T), and prolactin (PRL)) were determined. Plasma glucose was determined with the glucose hexokinase method (Cobas Integra 400 Plus, Roche Diagnostics, and Mannheim, Germany). Hormone profile is measured with electrochemiluminescence assays (ELECYS 2010 HITACHI, Roche Diagnostic, Germany).

Plasma concentrations of insulin were measured by chemi-luminescent immunoassay (Immulite One; BioDPC, Los Angeles, CA, USA). Insulin resistance was measured with HOMA-IR (homeostasis model assessment for insulin resistance) [18].

All the participants were subjected to careful ear examination to identify any abnormalities that may interfere with hearing such as a perforated tympanic membrane or other middle ear pathologies. All subjects had normal immittance audiometry results. Each participant was tested with Low Frequency Audiometry (LFA); 125 Hz to 2 kHz, High Frequency Audiometry (HFA); 4 kHz to 8 kHz, and Extended High Frequency Audiometry (EHFA); 9 kHz to 20 kHz. Audiometry was performed by an expert audiologist blinded to the study.

All statistical analyses were performed using the SPSS for Windows, version 17.0. Nonparametric tests were used as the variable hearing threshold had abnormal distribution due to data dispersion and lack of distribution symmetry. After testing the normal distribution, comparisons between the groups were tested using the *t*-test. The chosen significance level was *P* < 0.05.

## 3. Results

Following through examination, otologic and audiometric evaluation, 40 patients with PCOS and 25 healthy controls were included in the final analysis. Two groups were comparable with regard to age and BMI. The mean age was 23.8  ±  4.6 years in PCOS group and 24.6  ±  4.8 years in control group. The mean BMIs were 25.9 ± 4.8 kg/m^2^ and 24.4 ± 3.8. Demographical and laboratory findings are shown in Tables 1 and 2.

A significant difference in LH, LH/FSH, Testosterone, CRP, fasting glucose, fasting insulin, and HOMA index was observed among the two groups (Table 2).

**Figure 1:**
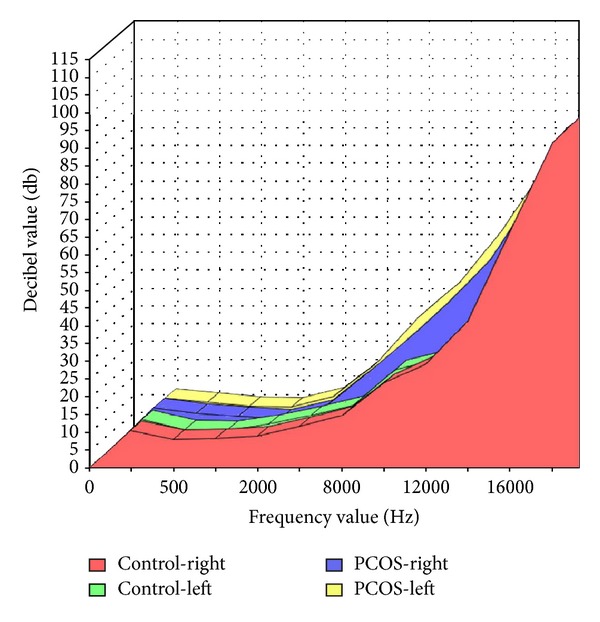
Comparison of hearing levels between groups.

The hearing thresholds for the left and right ears are described in Table 3. Although the hearing thresholds of the groups were similar at frequencies of 250, 500, 1000, 2000, and 4000 Hz, significant hearing loss was observed at frequencies of 8000, 10000, 12000, and 14000 Hz in PCOS group compared to controls (Figure 1). There was no statistically significant difference in tympanometric results between the two groups.

## 4. Discussion

To the best of our knowledge, this is the first study evaluating extended high frequency hearing loss in women with PCOS compared to healthy women. Routine audiometry is restricted to 125–8000 Hz frequencies. However, in diseases with endothelial damage, high frequencies are mostly affected in early stages. High frequency audiometry was introduced into clinical practice in 1960s [19].

In the present study, HOMA-I, CRP, LH, LH/FSH, testosterone, fasting glucose, and insulin levels were higher in women with PCOS. CRP is a circulating marker of the proinflammatory state in PCOS as evidenced by the 2-fold elevation in circulating CRP compared to controls. Similarly, in our study CRP was significantly higher in PCOS patients (*P* < 0.05). A meta-analysis of the most comparable studies indicates that circulating CRP is elevated in PCOS reflective of the chronic low-grade inflammation present in the disorder. They also found that elevated circulating CRP in PCOS is independent of obesity [13]. Although we could not demonstrate a direct correlation between CRP levels and hearing thresholds, chronic inflammation may play a role in the pathogenesis of hearing impairment in PCOS. Main causes of hearing loss seen in EHFA in PCOS patients may also be multifactorial.

Early signs of endothelial damage and increased cardiovascular disease risk have been previously described in PCOS patients [20]. Atherogenesis and endothelial dysfunction have been attributed to a number of biochemical and hormonal alterations in PCOS. Elevated mean platelet volume, white blood cell, D-Dimer, and androgen levels have been reported as potential indicators of risk factor for atherogenesis [21, 22].

EHFA with determination of air conduction thresholds in the frequency range of 8–20 kHz has been used in a number of studies in recent years [23]. Sensorineural hearing loss has been reported in a number of chronic systemic inflammatory disorders such as ankylosing spondylitis and rheumatoid arthritis, but exact cause of underlying pathology is still unknown [24, 25]. However, immunomediated vasculitis in the inner ear and ototoxic medication used for treatment was postulated as causative agents in some. Hearing losses in autoimmune diseases are reported to be mediated by vascular mechanisms [26].

Oghan and Coksuer firstly described high frequency (4000–8000 Hz) hearing loss in PCOS patients. They attributed this to the hyperandrogenism seen in the syndrome [16]. In the present study, we evaluated the hearing thresholds in EHF (8000–20000 Hz). We observed statistically significant difference in hearing thresholds between PCOS and control groups in 8000, 10000, 12000, and 14000 Hz. There were no significant differences in hearing thresholds comparing right to left ears at all frequencies (250–20000 Hz).

Despite certain limitations of our study, the results might be as preliminary findings for designing future studies on larger populations. A limitation of this study is small population, but strength of this study is evaluating the EHFA in all participants.

High frequencies are more sensitive to vascular damage caused by the disease. Insulin resistance, hyperandrogenemia, and elevated serum CRP as an inflammatory marker in PCOS could be the cause of hearing loss in especially extended high frequencies. Our data suggest that HFA and EHFA are more efficient in detecting early hearing loss compared to pure tone audiometry. Further studies are needed to help elucidate the mechanism behind hearing impairment in association with PCOS and to see whether the impairment of EHFA in these cases are progressive. It might be possible to prevent progression of hearing impairment by revealing underlying factors.

## Figures and Tables

**Table 1:** Demographic findings of subjects.

Groups	*N*	Mean	Standard deviation	*P*
Age control PCOS	25	24,6400	4,64471	0,465
	40	23,7843	4,83865	
BMI control PCOS	25	24,3960	3,81930	0,155
	40	25,9784	4,80293	

**Table 2:** Biochemical parameters of PCOS and control group.

	PCOS	Control	*P*
Fasting Glucose	85,2 ± 29,8	82,7 ± 37,3	**0,027**
Insulin	14,3 ± 7,8	4,9 ± 1,2	**0,028**
Homa-1	3,2 ± 2,7	1,6 ± 0,3	**0,020**
CRP	0,3 ± 0,4	0,1 ± 0,1	**0,016**
MPV	8,3 ± 0,9	8,1 ± 1,0	0,644
TSH	2,3 ± 1,1	2,2 ± 1,1	0,859
LH	9,5 ± 5,5	5,7 ± 2,7	**0,004**
FSH	5,1 ± 2,2	4,0 ± 1,9	**0,039**
LH/FSH	2,0 ± 1,1	1,4 ± 0,3	**0,045**
E2	96,9 ± 78,5	111,0 ± 97,6	0,324
Progesterone	5,3 ± 9,4	7,5 ± 7,5	0,494
PRL	15,5 ± 7,9	17,2 ± 8,4	0,403
Testosterone	0,4 ± 0,3	0,3 ± 0,1	**<0,0001**

**Table 3:** Hearing thresholds of PCOS and control group.

Frequency (Hz)		PCOS (Mean)	Control (Mean)	*P*
250	Right	12,9	11,7	0,907
	Left	12,1	11,7	0,318
500	Right	11,2	8,3	0,117
	Left	10,9	7,2	0,103
1000	Right	10,3	8,3	0,839
	Left	10,3	7,2	0,530
2000	Right	9,7	10	0,532
	Left	10,3	10	0,231
4000	Right	11	11,7	0,295
	Left	11,3	11,7	0,956
8000	Right	**22,1**	**12,8**	**0,02**
	Left	**21,9**	**12,2**	**<0,001**
10000	Right	**30,7**	**24,7**	**0,014**
	Left	**35**	**25,8**	**0,010**
12000	Right	**43,8**	**30,6**	**0,001**
	Left	**43,3**	**28,9**	**<0,001**
14000	Right	**56**	**41,4**	**0,028**
	Left	**63,8**	**44,2**	**0,004**
16000	Right	77,9	66,4	0,592
	Left	80,4	64,2	0,606
18000	Right	90	90	0,209
	Left	91,7	92,4	0,727
20000	Right	102,5	102,6	0,772
	Left	100,3	99,8	0,290
